# Association of severe malaria with cognitive and behavioural outcomes in low- and middle-income countries: a meta-analysis and systematic review

**DOI:** 10.1186/s12936-023-04653-9

**Published:** 2023-08-03

**Authors:** Andrew Sentoogo Ssemata, Ann Jacquelline Nakitende, Simon Kizito, Melissa R. Thomas, Sumaiya Islam, Paul Bangirana, Noeline Nakasujja, Ziyi Yang, Yunpeng Yu, Tuan M. Tran, Chandy C. John, Megan S. McHenry

**Affiliations:** 1 Department of Psychiatry, School of Medicine, Makerere University, P. O. Box 7072, Kampala, Uganda; 2 Department of Global Health and Development, London School of Hygiene and Tropical Medicine, London, UK; 3 Department of Mental Health and Community Psychology, School of Psychology, Makerere University, Kampala, Uganda; 4 Richard M. Fairbanks School of Public Health, Indiana University-Purdue University, Indianapolis, USA; 5 School of Medicine, City University of New York (CUNY), New York City, USA; 6 Mailman School of Public Health, Columbia University, New York City, USA; 7 Global Health Uganda, Kampala, Uganda; 8 Department of Biostatistics and Health Data Science, School of Medicine, Indiana University, Indianapolis, USA; 9 Division of Infectious Diseases, Department of Medicine, Indiana University School of Medicine, Indianapolis, IN USA; 10 Ryan White Center for Pediatric Infectious Disease and Global Health, Department of Pediatrics, Indiana University School of Medicine, Indianapolis, IN USA

## Abstract

**Background:**

Malaria affects 24 million children globally, resulting in nearly 500,000 child deaths annually in low- and middle-income countries (LMICs). Recent studies have provided evidence that severe malaria infection results in sustained impairment in cognition and behaviour among young children; however, a formal meta-analysis has not been published. The objective was to assess the association between severe malaria infection with cognitive and behavioural outcomes among children living in LMICs.

**Methods:**

Six online bibliographic databases were searched and reviewed in November 2022. Studies included involved children < 18 years of age living in LMICs with active or past severe malaria infection and measured cognitive and/or behaviour outcomes. The quality of studies was assessed. Definitions of severe malaria included cerebral malaria, severe malarial anaemia, and author-defined severe malaria. Results from all studies were qualitatively summarized. For studies with relevant data on attention, learning, memory, language, internalizing behaviour and externalizing behaviour, results were pooled and a meta-analysis was performed. A random-effects model was used across included cohorts, yielding a standardized mean difference between the severe malaria group and control group.

**Results:**

Out of 3,803 initial records meeting the search criteria, 24 studies were included in the review, with data from 14 studies eligible for meta-analysis inclusion. Studies across sub-Saharan Africa assessed 11 cohorts of children from pre-school to school age. Of all the studies, composite measures of cognition were the most affected areas of development. Overall, attention, memory, and behavioural problems were domains most commonly found to have lower scores in children with severe malaria. Meta-analysis revealed that children with severe malaria had worse scores compared to children without malaria in attention (standardized mean difference (SMD) −0.68, 95% CI −1.26 to −0.10), memory (SMD −0.52, 95% CI −0.99 to −0.06), and externalizing behavioural problems (SMD 0.45, 95% CI 0.13–0.78).

**Conclusion:**

Severe malaria is associated with worse neuropsychological outcomes for children living in LMICs, specifically in attention, memory, and externalizing behaviours. More research is needed to identify the long-term implications of these findings. Further interventions are needed to prevent cognitive and behavioural problems after severe malaria infection.

*Trial Registration*: This systematic review was registered under PROSPERO: CRD42020154777.

**Supplementary Information:**

The online version contains supplementary material available at 10.1186/s12936-023-04653-9.

## Background

Despite progress towards mitigating malaria as a global health challenge, malaria disproportionally affects children in low and middle-income countries (LMICs). In 2020, there were over 625,000 malaria deaths globally, 96% of which occurred in sub-Saharan Africa [1]. Within this region, 50% of the deaths were in children under 5 years of age [1]. Children surviving malaria have increased risks of neurological, cognitive, and behavioural problems [2–6]. The long-term impacts of malaria infection, especially severe malaria, are exacerbated by other conditions common within LMICs, such as anaemia and undernutrition [4, 7].

Although there are significant reductions in global malaria-associated morbidity and mortality over the last decade [8, 9], progress has stalled [10], and children residing in malaria-endemic LMICs continue to suffer from the consequences of malaria infection [11]. In recent years, cognitive and behavioural assessments are increasingly performed in research settings among children in LMICs [12–16]. There is mounting evidence demonstrating an association between severe malaria infection and poor cognitive or behavioural outcomes. Many previous publications of these data have used mini-review methodology; few were able to establish the full extent of both cognitive and behavioural deficits [4, 7, 17].

This literature review aims to fill the gap in systematic data on the association of severe malaria infection with cognitive and behavioural outcomes among children living in LMICs. LMICs are highlighted in this review due to the continued endemicity of malaria, widely-varied quality of malaria care, and other risk factors for poor development among many of these countries [18, 19]. Within this systematic review and meta-analysis, the objectives were to determine the affected domains of cognition and behaviour and to quantify the impact of severe malaria on these cognitive and behavioural outcomes.

## Methods

This study was conducted and reported in accordance with the Preferred Reporting Items for Systematic Reviews and Meta-Analyses (PRISMA) guidelines [20]. The systematic review protocol was registered on the International Prospective Register of Systematic Reviews (PROSPERO; registration number: CRD42020154777) and published [21].

### Data sources and search strategy

Literature searches for studies conducted between 1920 and November 17, 2022 were performed using MEDLINE (via PubMed), Cumulative Index to Nursing and Allied Health (via EBSCO), PsycINFO (via EBSCO), Embase, and The Cochrane Central Register of Controlled Trials. For studies conducted between 1920 and February 2023, Google Scholar and other relevant websites (WHO, Malaria consortium and OpenGrey) were included for additional potentially eligible articles.

A cut off of 1920 was used, as a preliminary review of the literature suggested no relevancy in citations retrieved prior to this year. The search was limited to studies published in English and available in full text. Reviewers also viewed the bibliographies of pertinent studies to ensure all potentially eligible studies were included. The search strategy was developed by an experienced medical librarian at Indiana University and citation management was performed using EndNote [22]. The final search terms included: malaria (asymptomatic, uncomplicated, and severe); cognitive, behaviour, child development; cognition, child; among others. A full description of the search strategy is included in Additional file 1. Of note, the original search criteria were inclusive of all types of malaria. However, after the search was performed, it was decided that the original protocol would be amended to split the review into two; one on severe malaria and the other on non-severe malaria. The PROSPERO amendment was submitted on August 29, 2022, and published on December 7, 2022. The literature review for non-severe malaria outcomes will be submitted for publication separately.

### Eligibility criteria

To be included, studies needed to include the following: (1) population aged 0–18 years; (2) laboratory diagnosis of an active or recent (most recent malaria episode within the past 12 months) malaria infection during admission; (3) cognitive and/or behaviour outcomes measured using standardized psychological tools (questionnaire-based scales and/or neurocognitive assessments); (4) studies required a comparator or control group used with similar outcomes and (5) study setting was within a LMIC, as defined by 2020 World Bank criteria [23, 24]. Studies excluded were: (1) reviews, editorials, and case series with < 5 patients and case studies; (2) non-human studies; and (3) non-English studies. In the majority of included studies, children were diagnosed with severe malaria when they: (1) had *Plasmodoim falciparum* or asexual malarial parasites present on blood smear; (2) tested retinopathy positive; (3) were admitted to hospital with coma, inability to localize pain, and no other causes of coma and (4) had a Blantyre coma score = / < 2 or = / < 4. However, any author description of severe malaria, severe malarial anaemia, or cerebral malaria diagnosis was allowed.

### Study procedures and data extraction

Using a two-tier approach, two reviewers independently screened all titles and abstracts after the removal of duplicates. Thereafter, they independently reviewed full-text papers within the context of the eligibility criteria, and any discrepancies were resolved in consultation with a third reviewer. After screening the titles, abstracts, and full texts, data were extracted independently based on the eligibility criteria and guided by an ad-hoc developed Excel spreadsheet. The outcomes extracted were: study setting (e.g., authors, year, country, setting, length of follow-up); sample characteristics (e.g. age, gender, number of participants included); study design (e.g. randomized controlled trials, case control, cohort, cross-sectional) descriptions of how malaria, cognition, and behaviour are assessed (tests and cut-off values used); and study outcomes.

For studies included in the meta-analysis, mean scores and standard deviations were extracted for children with and without cerebral malaria within each defined cognitive and behavioural domain. When means and standard deviations were not available in the published study, the corresponding author was contacted for more information as appropriate.

### Quality assessment

Five authors independently assessed the methodological quality and bias risks of selected studies during data extraction by following a predefined criteria derived from the literature review [25]. The psychometric quality of the final selected articles was assessed using the National Institutes of Health (NIH) and National Heart, Lung and Blood Institute (NHLBI) study quality assessment tools [26]. The tool signals critical appraisals to judge the risk of potential bias, with a high risk of bias rated as poor quality (score of 1–4), a mixed risk of bias rated as fair quality (score of 5–9) and low risk of bias rated as good quality (score of 10–14).

### Data synthesis and analysis

The extracted data from all included studies were narratively synthesized for review. Because the results of a single cohort were often published in multiple publications or used in the results of more than one study [3, 27–44], studies are reported based on cohorts, in addition to independent articles, to avoid misrepresenting the number of children included within these studies. The information from the studies meeting inclusion criteria was summarized and described qualitatively. A meta-analysis was performed using the cognitive and behavioural domains for which three or more cohorts had measured outcomes. When multiple studies described similar outcomes in the same cohort [3, 27–44], the results from only the most recent publication were pulled for the meta-analysis. Of note, higher numeric scores on the behavioural measures indicated worse behaviours and lower scores on the cognitive measures indicated worse cognition.

Given the heterogeneity of the included cohorts and study methodologies, a random-effects model was adopted for the meta-analysis. Additionally, the included studies differed in their use of cognitive or behavioural assessment tools, and thus the group mean and standard deviation of assessment scores were collected for the severe malaria and comparative control group of each study. If the standard deviations were not available, the standard error or 95% confidence interval (CI) were pooled.

Some articles published data without a group mean or direct comparison between baseline assessment among the two groups; in these cases, the corresponding authors were contacted for additional information. Given the differing assessment tools, two biostatisticians standardized the assessment scores to yield an effect size Hedge’s *g* between the severe malaria group and the control group. A standardized mean difference and 95% CI were produced for each cognitive or behavioural domain: language, learning, memory, attention, and internalizing or externalizing behaviour. Outcomes reported by three or more cohorts are included to facilitate comparative analyses.

## Results

**Fig. 1:**
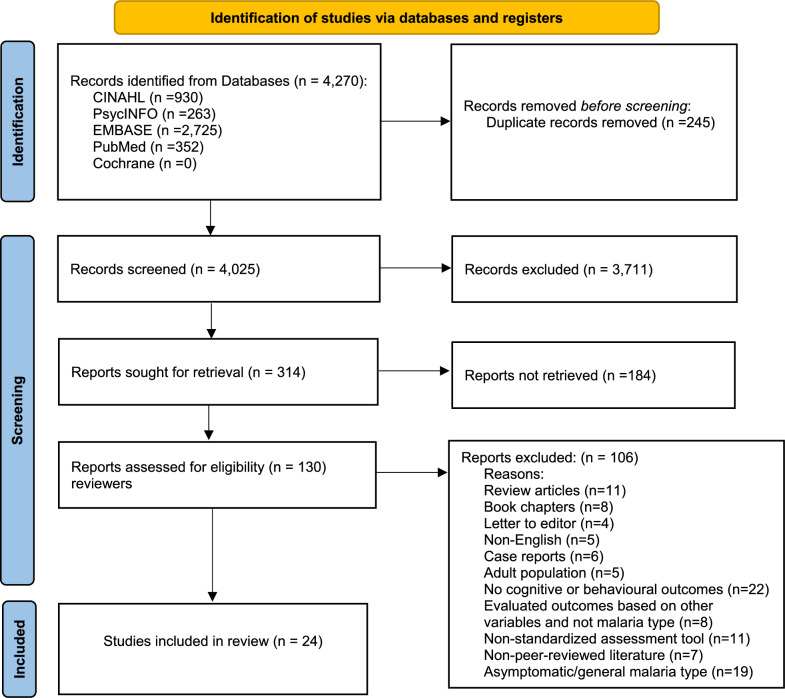
PRISMA 2020 Flow Diagram

The literature search yielded 4,270 titles. Of these, 314 were retrieved for full-text review, 24 were included in the systematic review, and 14 articles had data used within the meta-analysis (Fig. 1). Within the 24 studies included in the review, 11 distinct cohorts were studied (n = 4,143 participants). Of these, four cohorts were based in Uganda (n = 10 studies), three in Kenya (n = 8), two in Malawi (n = 4), one in Gambia (n = 1), and one in Senegal (n = 1).

The ages of children across the studies ranged from 18 months to 12 years of age. The most commonly used tests of cognition and behaviour were the Kaufman Assessment Battery for Children (KABC-II) (n = 9) and Achenbach Child Behavior Checklist (CBCL) (n = 5), respectively. Of the 11 cohorts, four described a complete cohort of cerebral malaria survivors, three cohorts evaluated a mix of malaria types among group of children, and two cohorts each (four articles total) evaluated children who had severe malaria anaemia or complicated seizures. No articles were judged as poor quality, eight were judged as fair, and 16 as good quality. The full study characteristics for each included article can be found in Additional file 2: Table S2.

### Narrative synthesis of included studies

#### Ugandan cohorts

Of the four Ugandan cohorts included in this study, all were based at Mulago Hospital in Kampala. Two prospective studies of children aged 5–12 years old recruited from 2003 to 2005 with different forms of malaria infection: children with cerebral malaria (n = 44), children with uncomplicated malaria (n = 54), and healthy children from the community (n = 89) [3, 27]. The first study followed the cohort up to 6 months after their malaria episode or enrollment, while the second expanded the follow-up to 24 months. Working memory, attention, and tactile-kinesthetic learning were assessed at baseline, 3-months, 6-months, and 24-months post-discharge. At 6 and 24 months, children with cerebral malaria had a 3.7-fold increased risk of having a cognitive deficit (i.e. a score of ≤ 2 standard deviations of the mean of community control children in any of the above three domains assessed) compared to controls (95% CI 1.3–10.7, P = 0.02; 95% CI 1.11–12.07, P = 0.03, respectively) [3, 27]. At 6 months post-hospital discharge, those with cerebral malaria had significant deficits measured in their working memory (P = 0.04) and attention (P = 0.005) [27]. At 2 years post-hospital discharge, only the difference in attention remained significant between children with cerebral malaria and the community controls [3].

A separate cohort of children < 5 years of age with cerebral malaria and severe malarial anaemia were assessed using the CBCL, Early Childhood Vigilance Test, the Color Object Association Test, and the Mullen Scales of Early Learning (MSEL) at 1 year after their malaria episode [38–40, 45]. The participants were recruited between November 2008 and January 2012. Across the baseline, 6-month, and 12-month assessments, children with cerebral malaria (n = 80–102) had significantly lower scores in various neurocognitive functions than controls (n = 61–64), including associative memory (P = 0.002), attention (P = 0.02), and all MSEL domains—fine and gross motor, visual reception, and receptive and expressive language skills (P < 0.001 for all) [38, 39]. Those with severe malarial anaemia (n = 86–102) showed differences from controls in attention (P = 0.08) and overall cognitive ability (P = 0.01) [38, 39].

Behaviourally, children with cerebral malaria (n = 173) in this < 5 years of age cohort had more mental health and behavioural disorders, mostly externalizing disorders (P = 0.01), compared to community controls (n = 108) (P = 0.99) [45]. Children surviving cerebral malaria (n = 122) and severe malarial anaemia (n = 130) reported significantly higher scores in internalizing (P = 0.04, 0.05, respectively) and externalizing problems (P = 0.001, 0.04, respectively) compared to healthy counterparts (n = 149) at 24 months [40]. Among children of the same age in a different cohort, those with severe malaria (n = 9 with cerebral malaria; n = 34 with malaria with seizures; n = 19 with malaria with impaired consciousness) had no differences in cognitive or attention scores compared to controls (n = 61), in exception of a significant difference in internalizing behaviours [37]. A more recent evaluation of older children from Mulago Hospital, aged 5–12 years and recruited between 2012 and 2016, observed worse behavioural scores and marginally lower cognitive scores among children with severe malaria (n = 37–150) compared to community controls (n = 87–150) [36, 43, 44].

#### Kenyan cohorts

Of the three Kenya cohorts described within eight studies, all were based at the Kilifi District Hospital on the eastern coast of the country. In one cohort, children aged 6–9 years with either cerebral malaria (n = 13–152) or severe malaria with complicated seizures (n = 156) were compared with a random sample of community children (n = 27–179) deemed unexposed—but likely living with mild malaria– on language assessments and the Rivermead Behavioral Memory Test [28–33]. All participants in this cohort were born between 1991 and 1995. Children with cerebral malaria were more likely to record impairments in multiple language domains than unexposed children across all six studies. Multiple studies found consistently reduced scores in language tests among the severe malaria with complicated seizures group that largely did not reach statistical significance [28–31, 33]; one study also identified significantly worse scores in behaviour within children with complicated seizures compared to controls (P = 0.05), and no differences in cognitive tests of memory or attention [31]. Persistent memory impairment, in contrast, was found to be common in both complicated severe malaria groups (cerebral malaria and severe malaria with seizures) across three studies [29, 32, 33].

Two additional cohorts consisting of severe malaria survivors from the Kilifi District Hospital were described in separate studies [46, 47]. One study shared similar population characteristics with the described cohort above, though patients were recruited at a different time point 10 years later, between 2004 and 2005 [46]. Among these children with severe malarial phenotypes (n = 30 with impaired consciousness; n = 15 with complicated seizures; n = 9 with prostration; n = 4 with seizures and fever) between 6 and 9 years of age, there were no differences across multiple tests of cognitive functioning and attention compared to controls, though differences in executive function domains of attention and vigilance for children with complex seizures were noted (P = 0.029) [46]. In a separate cohort, children with severe malaria who were > 9 months of age at admission (n = 87) performed worse on subtests of language (P = 0.02), attention (P = 0.03), and behaviour (P < 0.001) compared to age-matched controls for the Kilifi Assessment Battery within 42–70 months post-hospital discharge [47].

#### Malawian cohorts

Two cohorts of Malawian children presenting with retinopathy-positive cerebral malaria to Queen Elizabeth Central Hospital in Blantyre were assessed using the culturally validated Malawi Development Assessment Tool (MDAT) across four studies [34, 35, 41, 42]. Both cohorts consisted of cerebral malaria survivors (n = 49–104) age-matched with community controls (n = 60–117). One cohort assessed preschool (2–6 years of age) and school-aged (4–8 years of age) pairs every 3 months for up to 3 years between 2005 and 2007 [34, 35]. In the second cohort, children < 7 years of age were assessed every 6 months for up to 5 years following their hospital discharge between 2012 and 2014 [41, 42].

In the first cohort, children with cerebral malaria scored significantly lower in the language and motor domains of the MDAT, with slight differences in internalizing behaviours and no differences in the social scores [35]. A follow-up study 8 years later compared preschool and school-aged children across the MDAT, CBCL, KABC-II, and Tests of Variables of Attention; the only significant differences remained in global KABC-II domains (list of domains: sequential processing, simultaneous processing, learning, and planning) among school-aged children (P < 0.01 for all) [34]. In the second cohort, only malaria survivors < 5 years of age were delayed in motor, language, and social development (P < 0.03 for all), while there were no differences in cognitive function among between cases and controls > 5 years of age [41]. In a follow-up study, all children with cerebral malaria < 7 years of age had lower scores on a combined cognitive measure of the KABC-II and MDAT assessment (P < 0.001) and disruptive behaviour in BRIEF (P = 0.02) over a period of 12 months [42].

#### Senegalese and Gambian cohorts

Two additional studies describe cohorts in Senegal and Gambia. Among school-aged Senegalese children, those with cerebral malaria 6 years earlier (n = 29) performed worse on the KABC-II scales of sequential and simultaneous processing (P < 0.01, P < 0.05, respectively), as well as omission errors in the Tests of Variables of Attention (P < 0.05), than the comparative control group of children living with mild malaria (n = 29) [48]. However, a study of matched Gambian children 5–8 years of age found that although children with a previous cerebral malaria episode 3 years earlier (n = 40) had lower mean scores across multiple cognitive and motor tests, none reached statistical significance [49].

### Meta-analysis of cognitive and behavioural outcomes

**Fig. 2:**
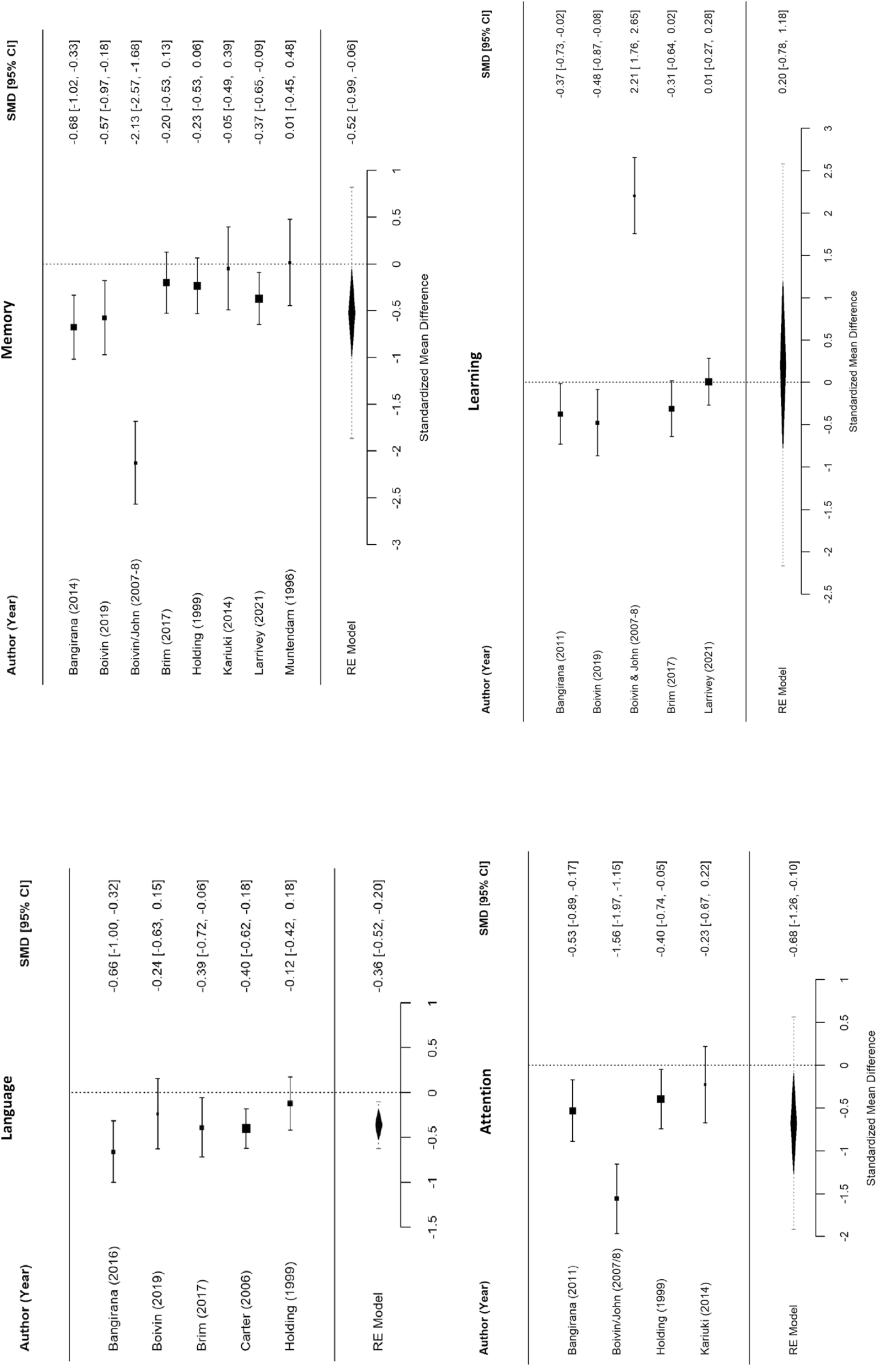
Meta-analysis of cognitive outcomes in children with severe malaria versus uninfected children. (Note: for all cognitive scores, lower scores mean worse cognition.)

**Fig. 3:**
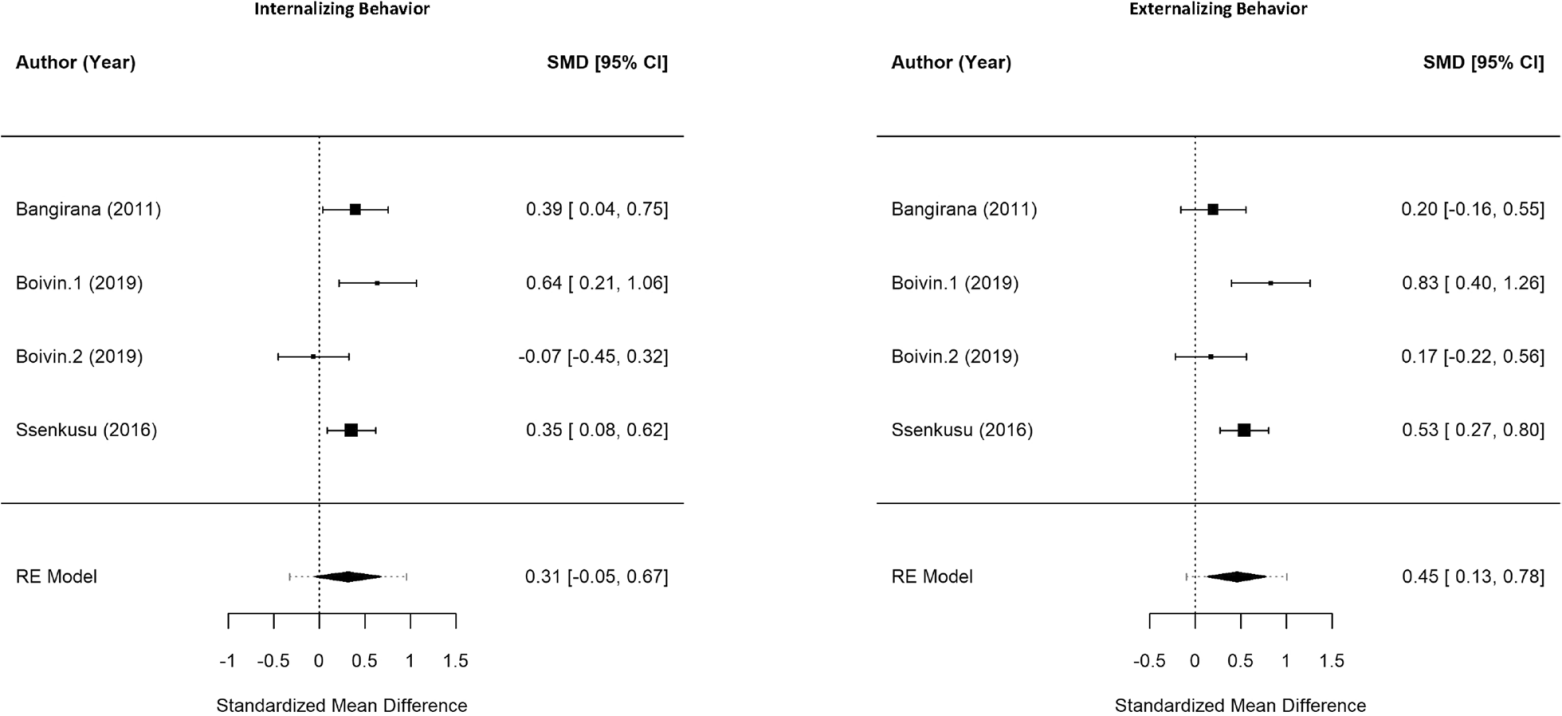
Meta-analysis of behavioural outcomes in children with severe malaria versus uninfected children. (Note: for behavioural scores, higher CBCL scores indicate worse behaviour.)

Approximately 79.17% (19/24) of studies in the overall review indicated poor performance among children with cerebral malaria in at least one test or domain of cognitive/behavioural outcomes, although the meta-analysis showed that only 57.14% (8/14) of included studies resulted in a significant difference among cognitive outcomes in the selected domains [3, 27, 28, 34, 37, 39, 41, 43, 47]. Overall, children with severe malaria performed worse in both behavioural domains and all cognitive domains, except learning, when compared to controls (Figs. 2, 3).

Memory (SMD −0.52, 95% CI −0.99 to −0.06) and attention (SMD −0.68, 95% CI −1.26 to −0.10) were significantly affected by severe malaria. Children with severe malaria also significantly performed worse in tests of language (SMD −0.36, 95% CI −0.52 to −0.20) compared to the control group. Interestingly, children with severe malaria showed significantly worse scores in externalizing behavioural problems (SMD 0.45, 95% CI 0.13–0.78), but not in internalizing behavioural problems (SMD 0.31, 95% CI −0.05 to 0.67).

## Discussion

In children, severe malaria is associated with a range of worse cognitive and behavioural outcomes, most significantly in memory, attention, language, and externalizing behavioural problems. Although early cognitive deficits following hospital discharge from severe malaria episodes can improve overtime, some studies suggested that attention, memory, and behavioural problems may persist for up to 2 years following the malaria episode [3, 29, 32, 33, 42, 47]. These outcomes demonstrate the potential impact of severe malaria infection on reduced school performance and learning capacity among children living in LMICs. The review provides useful insights to quantify the potential impact of childhood severe malaria on cognitive or behavioural problems [6].

Based on the meta-analysis, there is a clear linkage between worse attention, memory, language, and externalizing behavioural outcomes for children with past severe malaria. Scores within these cognitive and behavioural domains are strongly associated with school performance in early childhood, particularly when deficits occur simultaneously [50]. A systematic review in 2022 suggests that externalizing behavioural problems alone have mixed results for lower academic performance [51], but that the combination of inattention accompanying behavioural problems contributes to consistently low performance before secondary school [52]. Further, there is strong evidence that low attention independently contributes to lower chances of graduating from secondary school and worse academic performance [52]. Working memory and inattention delays appear in conjunction with poor development of academic skills, particularly in mathematics and language development [53–55], an additional aspect of cognition affected by severe malaria. Inhibition, attention, and memory make up components of executive functioning, a key aspect of a child’s cognitive development. Worse performance in executive functions, even in early childhood, is strongly associated with lower educational attainment across the life course [56]. This relationship is especially detrimental in LMICs, where children already experience many risk factors for poor cognition, academic achievement, and economic productivity, such as extreme poverty, malnutrition, exposure to violence, and recurrent illness [57]. Therefore, the effects of severe malaria may exacerbate disparities of human capital and economic development in LMICs that already experience a high burden of malaria and cognitive impacts [56, 58].

While this systematic review and meta-analysis provides useful insights into these cognitive domains, additional studies are needed to better assess the effect of severe malaria on the long-term consequences of cognition and behaviour. For instance, the number of episodes of severe malarial infection and its association with cognition needs to be further explored to determine the specific pathological mechanisms [59]. Future research would benefit by differentiating the cognitive and behavioural outcomes of severe malaria subtypes: cerebral malaria, severe malarial anaemia, severe malaria with seizures, and severe malaria. In this review, only four articles differentiated severe malarial anaemia from cerebral malaria [38–40, 45]. From these preliminary comparisons, children with severe malarial anaemia have less severe outcomes than those with cerebral malaria; however, these data are not able to be fully explored in the scope of this review, as only two cohorts existed across included studies [38–40, 45]. Future research would serve to evaluate severe malaria types further and beyond grouping them together.

The current review indicates that the current data of cognitive and behavioural effects from severe malaria are limited within study populations of sub-Saharan Africa. The included studies take place across Uganda, Kenya, Malawi, Senegal, and Gambia, representing some of the more malaria-burdened countries globally [60]. However, all of the included cohorts were centralized within one area, such the eastern region of Kenya or the capitol of Uganda. This may limit the data on LMIC settings, which are also affected by outcomes of severe malaria infection. Particularly, the four African countries that account for over 50% of all global malaria deaths—Nigeria, the Democratic Republic of Congo, United Republic of Tanzania, and Niger—were not included in any assessments of severe malaria infection and cognitive outcomes in this review [11]. Further, the main malaria species implicated as the causative agent across included studies was *P. falciparum*, given its overwhelming prevalence in sub-Saharan Africa and potential for severe acute disease in young children. Given the limits of these data, it remains unclear whether other malaria species, such as *Plasmodium malariae* or *Plasmodium vivax*—which are prevalent in Asia, Latin America, and some parts of Africa—may also affect cognition.

While this systematic review compared children with severe malaria with those who were presumed to be uninfected, some of the studies also included a group of children with uncomplicated malaria for analysis [3, 27]. Within these studies, children surviving severe malaria were still found to have worse scores in working memory, attention, and learning with a large margin of difference [3, 27]. These results demonstrate clear outcomes of worse cognitive performance, which incorporate many elements of development, in children who were diagnosed with cerebral malaria. The period of early childhood is critical for achieving the full neurodevelopmental potential of children living in LMICs, who already face many barriers to academic achievement and economic mobility in their adulthood [57, 61]. A previous systematic review indicated that microvascular obstruction, microcirculatory impairment, and tissue hypoxia may be directly involved in the pathophysiology of severe malaria [62]. These brain structures are central to the production of excessive inflammatory cytokines, such as TNF, and could influence local concentrations of pro-inflammatory cytokines in the brain [63]. As neurological complications specific to cerebral malaria are being understood, it is clear that axonal injury influences the cognitive impairment of children with severe malarial disease and inhibits their critical period of neurodevelopment [64].

Updated guidelines from the World Health Organization reintroduced critical strategies for preventing and eliminating malaria across LMICs, following COVID-related disruptions in malaria prevention [65]. Malaria transmission continues to heavily impact low-resourced settings despite these efforts, and global malaria elimination is still far from reached. Until then, it is key to reduce childhood incidence of malaria and mitigate the effects of children who are impacted by severe malaria. The findings of this review may contribute to guiding the development of interventions and improving strategies towards offering high-quality interventions for children in LMICs. Although researchers are developing tools for neurocognitive assessment among children in LMICs, there are limited evidenced-based interventions for children with neurocognitive deficits in these settings. Future research may assess the benefit of malaria prevention and rehabilitation interventions for the improvement of cognitive and behavioural outcomes.

### Limitations

This systematic review considerably expands on previous evidence and advances the knowledge of the effects of severe malaria on children’s neuropsychological function. A variety of study designs with well-characterized participants are included in the analysis, beyond the existing mini reviews on malaria and cognitive and behavioural function [7, 17]. Additionally, there are other ways of measuring the impact of malaria on the brain, beyond what this review encompasses. The relative homogeneity of included assessment tools in the meta-analysis may have excluded other measurements of neuro-functioning, such as electroencephalography and neuroimaging [66–68]. Other methods for evaluating neurophysiology and neurological performance should be considered for future studies to better understand the mechanisms and potential areas for intervention. Of note, none of the cognitive tools in the review had been used to assess the behavioural and cognitive deficits before the onset of malaria. Only five studies included information of malaria chemoprevention [3, 27, 29, 31, 48], limiting the review’s ability to provide data on the impact of treatment on cognition or behaviour, as well as the impact of co-infections such as HIV or anaemia, which were not consistently evaluated across the included studies.

An additional limitation is the inclusion of multiple publications from the same cohort; it is unclear whether specific participants were subsequently included in multiple cohorts based on the age of enrollment. However, important points are uncovered for interpreting data from severe malaria studies. Only 11 cohorts were assessed from four countries, all of whom have a predominant strain of malaria present. Furthermore, the duration of follow up was quite variable, so it is difficult to quantify how long these effects persist or when follow-up should take place, if these children are to be evaluated post-severe malaria episode.

## Conclusions

Severe malaria contributes to the poor cognitive and behavioural performance of children in LMICs. Worse cognitive scores post-malaria represent a significant global health challenge to reduced school achievement and delayed physical development. The analysis demonstrates that severe malaria in LMICs affects both neurodevelopment and behaviour in children, with long-lasting implications. The findings of this review also indicate that a limited number of cohorts have contributed to the current evidence regarding the malaria effects on neurodevelopment among children in LMICs. Children living in LMICs are vulnerable to cognitive and behavioural impairment associated with persistent illnesses, such as malaria, and other mechanisms, such as poverty and parental education [69]. Until population-based improvements are made to reduce risk factors for poor development, early identification and rehabilitation of cognitive and behavioural deficits among children surviving malaria is an important aspect of care to alleviate further decline in performance, and allow them to reach their full potential.

### Supplementary Information

**Additional file 1.** Search strategy.

**Additional file 2.** Characteristics and quality of the included studies. Abbreviations: SMA, Severe malarial anemia; BCS, Blantyre Coma Scale; KABC-II, Kaufman Assessment Battery for Children-Second Edition; BRIEF, Behavior Rating Inventory for Executive Function; CBCL, Achenbach Child Behavior Checklist; MDAT, Malawi Developmental Assessment Tool; MSEL, Mullen Scales of Early Learning; TOVA, Test of Variables of Attention; AMD, Adjusted mean difference; RR, Risk ratio; TPT, Tactual Performance Test.

## Data Availability

All data generated or analysed during this study are published in this article.
