# Supplementary material for: Association of severe malaria with cognitive and behavioural outcomes in low- and middle-income countries: a meta-analysis and systematic review
Source: Malar J. 2023 Aug 3;22:227. doi: 10.1186/s12936-023-04653-9 (PMC10401769; doi:10.1186/s12936-023-04653-9)
Supplement: Supplementary file 1 — Additional file 1. Search strategy. [file 12936_2023_4653_MOESM1_ESM.docx]

**Additional Table 1.**

**Search strategy**

("cognitive"[All Fields] AND (functions [All Fields] OR function [All Fields] OR functioning [All Fields])) OR (neurocognitive[All Fields] OR neurocognitively[All Fields] OR neurocognitives[All Fields] OR neurocognition[All Fields] OR "cognitive"[All Fields] OR cognitives[All Fields] OR cognitively[All Fields] OR "cognition"[MeSH Terms] OR "cognition"[MeSH Terms] OR "cognition"[All Fields] OR "cognition disorders"[MeSH Terms] OR ("cognition disorders"[MeSH Terms] OR ("cognition"[All Fields] AND "disorders"[All Fields])) OR "cognition disorders"[All Fields] OR "child development"[MeSH Terms] OR "child development"[Title/Abstract] OR "child behaviour disorders"[MeSH Terms] OR "child behaviour"[MeSH Terms]) AND ("malaria"[MeSH Terms] OR "malaria"[All Fields])

**Embase**

(('mental disease' OR 'cognition' OR 'behaviour disorder' OR 'developmental disorder' OR 'child development' OR 'neurological disorder' OR 'neurodevelopment' OR 'executive function' OR 'language') AND ('malaria' OR '*falciparum*' OR 'remittent fever') OR 'malaria infection' OR '*plasmodium*') AND ([adolescent]/lim OR [child]/lim OR [infant]/lim OR [newborn]/lim OR [preschool]/lim OR [school]/lim).
