# Supplementary material for: Association of severe malaria with cognitive and behavioural outcomes in low- and middle-income countries: a meta-analysis and systematic review
Source: Malar J. 2023 Aug 3;22:227. doi: 10.1186/s12936-023-04653-9 (PMC10401769; doi:10.1186/s12936-023-04653-9)
Supplement: Supplementary file 2 — Additional file 2. Characteristics and quality of the included studies. Abbreviations: SMA, Severe malarial anemia; BCS, Blantyre Coma Scale; KABC-II, Kaufman Assessment Battery for Children-Second Edition; BRIEF, Behavior Rating Inventory for Executive Function; CBCL, Achenbach Child Behavior Checklist; MDAT, Malawi Developmental Assessment Tool; MSEL, Mullen Scales of Early Learning; TOVA, Test of Variables of Attention; AMD, Adjusted mean difference; RR, Risk ratio; TPT, Tactual Performance Test. [file 12936_2023_4653_MOESM2_ESM.docx]

| Cohort | Author (Year) | Country | Study Design | Age Range of Participants | Characteristics of Study Cohort | Method of Malaria Diagnosis | Time between Malaria Infection and Cognitive Testing | Cognitive Tests Used | Results | Quality |
| --- | --- | --- | --- | --- | --- | --- | --- | --- | --- | --- |
| Cohort 1 | Miller (2022) | Uganda | Secondary analysis of a randomized controlled trial | 5-12 years of age, study activities 2012-2016, performed in Kampala | n= 74 severe malaria survivors  n= 87 not infected | Of children with severe malaria, those with (1) Blantyre Coma Scale (BCS) = 2 or Glasgow Coma Scale score of 8; (2) Plasmodium falciparum on blood smear; (3) No other cause of coma; and (4) positive retinopathy were diagnosed with cerebral malaria. | Evaluated at least two years after discharge from the hospital | School reports (developed by Ugandan Ministry of Education and Sports)  Kaufman Assessment Battery for Children-Second Edition (KABC-II): Non-Verbal Index | No significant differences were found in the KABC-II Non-Verbal Index or assessments of academic performance. | Fair |
|  | Larrivey (2021) |  |  |  | n= 37 cerebral malaria survivors  n= 64 with severe malarial anemia (SMA)  n= 100 not infected |  |  | KABC-II: Mental Processing Index, Sequential Processing Scale, Simultaneous Processing Scale, Learning Ability Scale, Planning | No differences seen between cerebral malaria and SMA, so groups were combined. For the Sequential Processing Domain score, all of the malaria group performed worse than the non-malaria children (P = 0.009). Mental Processing Index scores trended lower (i.e. worse) among malaria group compared to the non-malaria group (P = 0.073). | Good |
|  | Boivin (2019a) |  |  |  | n= 150 with severe malaria  n= 150 not infected |  |  | KABC-II: sequential and simultaneous processing  Behavior Rating Inventory for Executive Function (BRIEF)  Achenbach Child Behavior Checklist (CBCL): internalizing, externalizing, and total symptoms | Children with severe malaria trended towards worse scores on all KABC-II domains and the CBCL compared to uninfected children. | Good |
| Cohort 2 | Muntendam (1996) | Gambia | Cross-Sectional Case-Control | 5.4-8.5 years of age, study activities 1988-1991 | n= 40 with cerebral malaria  n= 40 community controls, matched on age, sex, ethnic origin, school type, and socioeconomic status. | Cerebral malaria was defined as (1) BCS ≤ 2; (2) asexual forms of *P. falciparum* on blood smear | Mean time was 3.4 years (1.3 SD) | Intellectual Development Tests: Classification; Word-object association; Exclusion; Syllable recall; Visual memory  Sensory-Motor Development: Pinboard; Mazes; Balls; Simple reaction time test; Choice reaction time test; Tapping test; Balance | Children with malaria trended to perform worse on balance tasks compared to unexposed controls (P = 0.05). No other significant differences found. | Fair |
| Cohort 3 | Langfitt (2019) | Malawi | Longitudinal Case-Control | Mean age at assessment was 48.6 months, study activities 2012-2014 | n= 85 with cerebral malaria  n=100 matched-hospitalized controls | Cerebral malaria was defined as (1) BCS ≤ 2; (2) *P. falciparum* on blood smear; (3) no other known cause of coma; (4) Retinopathy positive | 1 month, 6 month, and 12 month follow-ups | Children <5 years were assessed with the Malawi Developmental Assessment Tool (MDAT).  Children =/< 5 years were assessed by the KABC-II.  All caregivers of children >/= 2 year had a BRIEF performed. | Developmental impairment (P < 0.001) and disruptive behavior (P = 0.02) were significantly worse in malaria patients compared to the control group across all time points. | Fair |
|  | Brim (2017) |  |  |  | n=104 with cerebral malaria  n=117 matched- hospitalized controls |  | 1 month follow-up |  | Cases performed worse on MDAT scores within gross motor, fine motor, language, and social domains (all P <0.02 or lower). No differences were found on KABC-II or BRIEF assessments between the groups. | Fair |
| Cohort 4 | Bangirana (2014) | Uganda | Longitudinal Cohort | 18 months- 5 years of age, study activities 2008-2012 | n=80 children with cerebral malaria  n=86 with SMA  n=61 community controls | Cerebral malaria was defined as (1) BCS ≤ 2; (2) *P. falciparum* on blood smear; and (3) no other known cause of coma  SMA was defined as the presence of *P falciparum* on blood  smears in children with a hemoglobin  level ≤5 g/dL. | Tested one week after hospital discharge or at enrollment, then 6 and 12 months after enrollment | Overall cognitive ability- summative score of Mullen Scales of Early Learning (MSEL): fine motor visual reception, receptive language, and expressive language)  Associative memory  (Color Object Association Test)  Attention  (Early Childhood Vigilance Test) | When comparing adjusted scores between groups using all 3 testing time points, children with cerebral malaria had worse scores than controls in overall cognitive ability, attention, and associative memory (P <0.001 for all).  Children with SMA had worse scores compared to community controls in cognitive ability (P=0.01), but not attention or associative memory. | Good |
|  | Bangirana (2016) |  |  | 18 months-4 years of age, study activities 2008-2012 |  |  |  | MSEL: fine motor, gross motor, visual reception, receptive language, and expressive language | Across all 3 time points, children with cerebral malaria had worse scores in all MSEL neurocognitive abilities than the community controls (P < 0.005 for all).  Children with SMA had significantly worse scores than controls for visual reception (P=0.02), receptive language (P=0.03), and expressive language (P=0.04). | Good |
|  | Ssenkusu (2016) |  |  | 18 months-12 years of age, study activities 2008-2014 | n= 122 with cerebral malaria at baseline  n= 80 with cerebral malaria at 24 months  n= 130 with SMA at baseline  n= 81 with SMA at 24 months  n= 149 not infected at baseline  n= 90 not infected at 24 months |  | Tested one week after hospital discharge or at enrollment, 6 months, 12 months, and months after baseline enrolment | CBCL: internalizing behavior, externalizing behavior, and total symptoms. Higher scores indicated worse behavior. | For all visits combined, children with cerebral malaria and SMA had worse CBCL z-scores than uninfected children for internalizing (P= .01, P <0.001, respectively), externalizing (P <0.001, P= 0.003, respectively), and total problems (P <0.001 for both).  Children with SMA additionally scored worse than controls on every subtest at both the 12- and 24- month visits. | Good |
|  | Idro (2016) |  | Longitudinal | 18 months-12 years of age, diagnosed with severe malaria from April 2012 to December 2014. | n= 173 with cerebral malaria  n= 99 with severe malaria anemia  n=108 community controls | Cerebral malaria was defined as (1) BCS ≤ 2; (2) *P. falciparum* on blood smear; and (3) no other known cause of coma  SMA was defined as  the presence of *P falciparum* on blood  smears in children with a hemoglobin  level ≤5 g/dL. | >6 months after severe malaria diagnosis | Behavior  (Strengths and Difficulties Questionnaires)  Mini-International Neuro-Psychiatric Interview for Children and Adolescents (performed only for those with a SDQ score of ≥17) | Children with cerebral malaria had higher rates of abnormal scores for emotion (p=0.09), conduct (p=0.03), hyperactivity (p=0.004) and total difficulties (p=0.02) compared to community controls. Children with severe malaria anemia had higher rates of abnormal scores for conduct (p=0.04) compared to community controls. | Good |
| Cohort 5 | Boivin (2011) | Malawi | Retrospective case-control | 1.47-6.28 years, study activities 2005-2007 | n= 83 with cerebral malaria with retinopathy  n= 95 matched- hospitalized controls | For cerebral malaria: BCS ≤2, positive blood smear, no other known cause of coma, and retinopathy positive | 1–40 months | MDAT: gross motor, fine motor, language, social skills, total development (sum of scores)  CBCL: internalizing and externalizing symptoms | Children with cerebral malaria scored significantly worse in all MDAT domains, except social skills, and experienced more internalizing and externalizing problems (P= 0.30, P= 0.14, respectively) than the control group. | Fair |
|  | Boivin (2019b) |  |  | Mean age was 4 years (range 1.92-6.83) of age at preschool assessment  Mean age was 6.5 years (range 3.67-8) at follow-up assessment | n= 49 with cerebral malaria  n= 56 not infected | For cerebral malaria: BCS≤ 2, positive blood smear, no other known cause of coma, and retinopathy positive | Mean time was 17.88 months for the preschool assessment and 46.62 months for the school assessment. | Preschool-age children were assessed with the MDAT. School-age children were assessed with KABC-II and TOVA.  CBCL was used in both age groups. | While the case and control groups did not differ in scores of the MDAT, CBCL or TOVA, children with cerebral malaria did score significantly worse on all global KABC-II domains (all P <0.01 or lower). | Good |
| Cohort 6 | Carter (2006) | Kenya | Cohort | 6-9 years of age, born between 1991-1995 | n= 152 with cerebral malaria  n=156 malaria with complicated seizures  n= 179 not infected | Cerebral malaria defined as positive blood smear for malaria and BCS ≤ 2 for 4+ hours.  Malaria with complicated seizures defined as positive blood smear for malaria and prolonged seizures >30 minutes or 2+ seizures in 24 hours. | Minimum time was 20 months and maximum time was 112 months. | Speech and language assessments  Memory (Rivermead Behavioural Memory Test for Children),  non-verbal functioning (construction task), attention (visual search), and parental rating of behavioral problems (Kilifi Assessment Battery- adapted from the Vineland Adaptive Behavior Scales and Behavior Screening Questionnaire).  Impairment defined as an impairment score >2 for language, and standardized ability levels for cognitive abilities at <2 standard deviations from the mean of control group. | Eighteen (11.8%) of the cerebral malaria group, 14 (9%) of the malaria with seizures group, and four (2.2%) of the unexposed group were found to have a language impairment. Odds of having a speech and language impairment was increased in children with cerebral malaria (OR 3.68, 95%CI: 1.09-12.4) and trended high in children with malaria with seizures (OR 3.12, 95%CI: 0.9-10.8) compared to those children without malaria. | Fair |
|  | Carter (2005a) |  |  |  |  |  |  |  | 24% of the malaria group had at least one impairment in the major domains assessed, most commonly in the speech and language domain, compared with 10% of the control group. The cerebral malaria group had a higher proportion of multiple impairments compared to those with malaria with seizures. | Good |
|  | Carter (2005b) |  |  |  |  |  |  |  | Children with cerebral malaria performed worse or had increased odds of performing worse compared to the unexposed group in tests of higher-level language (adjusted mean difference (AMD) -1.63, 95%CI: -2.99 to -0.27), vocabulary (AMD -0.02, 95%CI: -0.04 to -0.01), pragmatics (OR 2.81, 95% CI: 1.04 to 7.6) and non-verbal functioning (AMD -0.33, 95%CI: -0.61 to -0.06).  The malaria with seizures group had reduced scores and higher odds of worse scores compared to the controls in phonology (OR 2.74, 95% CI: 1.26 to 5.95), pragmatics (OR 3.23, 95% CI:1.2 to 8.71) and behavior (OR 1.8, 95% CI: 1.0 to 3.23). This group scored higher than controls in syntax (AMD 1.26, 95% CI: 0.05 to 2.48). | Good |
|  | Idro (2006) |  |  |  | n= 143 with cerebral malaria  n= 179 not infected |  |  |  | Children with cerebral malaria were more likely to be impaired in memory (Risk ratio (RR) 5.0, 95% CI: 1.7 to 14.5), motor (RR 3.5, 95% CI: 1.3 to 9.5), behavior (RR 1.7, 95% CI: 0.4 to 7.3) and language functions than controls (RR 5.0, 95% CI: 1.7 to 14.5) compared to controls. | Good |
|  | Kihara (2009) |  |  |  | n= 152 with cerebral malaria  n= 156 malaria with complicated seizures (severe malaria)  n= 179 not infected |  |  | Speech and language assessment: receptive vocabulary and expressive language  Kilifi Creek Behavioural Memory Test for Children: everyday memory, recall, recognition, and prospective memory | When compared to unexposed children, those with cerebral malaria performed worse for recall (P=0.024), recognition memory (P=0.001), receptive vocabulary (P= 0.002), and expressive language (P = 0.002). When compared to children with malaria with seizures, those with cerebral malaria performed worse in everyday memory (P =0.002), recognition (P <0.001), and expressive language (P=0.013). Those with malaria with seizures did not perform significantly worse than those unexposed to malaria. | Good |
|  | Carter (2003) |  | Case-control | 7-10 years of age, born between 1990-1991 | n= 25 with severe malaria, including cerebral malaria (n=13) and malaria with seizures (n=12)  n= 27 not infected |  |  | Speech and language assessments: comprehension, syntax, lexical semantics, higher level language, and pragmatics | Children with severe malaria had significantly lower scores in comprehension, syntax, and lexical domains than uninfected children (all P= 0.02 or lower). | Fair |
| Cohort 7 | Boivin (2002) | Senegal | Case-control | 5-12 years of age | n= 29 with cerebral malaria    n= 29 with mild malaria | For cerebral malaria: positive blood smear, meeting World Health Organization 1986 criteria but extending coma time requirement to ≥12 hours, no response to antimalarial treatment in 24 hours.  For mild malaria: response to antimalarial treatment, 1-3 suspected episodes of malaria without fever or coma. | Mean time was 6 years. Average age at first cerebral malaria episode was 3.4 years. | KABC-II: global processing scales, sequential processing, simultaneous processing  TOVA: omission errors, commission errors, correct response time, response time for variability, and signal detection D prime | Children with cerebral malaria performed worse than children with mild malaria for percentage of TOVA omission errors (P < 0.001) and within multiple subscales of the KABC-II global, sequential, and simultaneous processing scales (P <.05, respectively). | Good |
| Cohort 8 | Holding (1999) | Kenya | Matched pairs design, matched on age, gender, and mother tongue | Mean age at assessment was 79.7 months (range 10-42 months) | n= 87 severe malaria survivors  n= 87 not infected | Modified BCS ≤4, cannot localize pain.  Method of malaria diagnosis not defined. | 42-70 months | Kilifi Assessment Battery: including tests on information-processing skills, achievement, visuo-motor speed, attention, planning, and language  Behavioral observation and assessment | When compared to controls, severe malaria survivors had worse scores in the articulation and syntax subtest of language (P= 0.02 for both), attention (P= 0.03), and behavioural observation outcomes by parent assessment (P <0.001). | Good |
| Cohort 9 | Kariuki (2014) | Kenya | Cohort | Interquartile range for malaria group was 6-7, for unexposed group was 6-8 | n= 58 with severe malaria (n=15 with complex seizures)  n= 56 not infected | BCS ≤2, complex symptomatic seizures, prostration, or seizures with fever, all with the diagnosis.  Method of malaria diagnosis not defined. | 1-2 years after discharge from hospital | Memory and attention (Self-Ordered Pointing Test; Computerized visual search task)  Executive function  (Contingency Naming Test)  Attention (Test of Everyday Attention for Children) | No differences in assessment performance between the case and control groups. However, sub analyses show worse scores in vigilance test (p=0.006) and attention (p=0.019) in children with malaria with complex seizures group (n= 15) when compared to unexposed group, even after adjusting for confounders. | Good |
| Cohort 10 | Boivin (2007) | Uganda | Cohort | 5-12 years of age, study activities 2003-2005 | n= 44 with cerebral malaria  n= 54 with uncomplicated malaria  n= 89 without infection | Cerebral malaria defined as (1) BCS ≤ 2 or Glasgow Coma Score ≤8; (2) P. falciparum on blood smear; and (3) no other known cause of coma  Uncomplicated malaria defined as (1) P. falciparum on blood smear; (2) not meeting World Health Organization criterium of severe malaria; and (3) presence of clinical symptoms such as fever, chills, vomiting, headache | At the time of hospital discharge or initial enrollment, 3 months, and 6 months later | KABC-II: simultaneous processing, sequential processing  TOVA: omission error rates, commission error rates, correct signal response time variability, d′ signal detection  Tactual Performance Test (TPT)  Cognitive deficit defined as z-score < -2 for memory and attention, and z-score ≥2 for learning | At each timepoint, children with cerebral malaria had an increased risk of a cognitive deficit in at least one domain compared with uninfected children, with 6 months carrying the greatest frequency of impairments (95% CI: 1.3-10.7, p=0.02) and a 3.7-fold increased risk of cognitive deficit. After 6 months, children with cerebral malaria had significant deficits measured in their working memory (p=0.04) and attention (p=0.005). At the time of discharge, children with cerebral malaria had more TOVA impairment scores than uninfected children, but there were no significant differences for TOVA or the TPT at other timepoints. | Good |
|  |  |  |  |  |  |  |  |  |  |  |
|  | John (2008) |  |  | 5-12 years of age, study activities 2003-July 2006 |  |  | At time of hospital discharge  or initial enrollment, 6 months later, 2 years later |  | Following 2 years since their hospital discharge, children with cerebral malaria had a 3.67-fold higher risk of a cognitive deficit compared to uninfected children. Only the differences in deficits in attention remained significant between children with cerebral malaria and uninfected children (p=0.005). | Good |
|  |  |  |  |  |  |  |  |  |  |  |
| Cohort 11 | Bangirana (2011) | Uganda | Prospective case-control | 5-12 years of age, study activities 2/2008-10/2010 | n= 62 with malaria and neurological involvement (n=9 with cerebral malaria, n= 34 malaria with seizures, n= 19 malaria with impaired consciousness)  n= 61 community controls | Positive blood smear  Prolonged seizures >15 minutes  Glasgow Coma Scale ≤ 14 | 3 months | KABC-II: working memory, visual spatial ability, learning, and reasoning  Test of Variables of Attention (TOVA): signal detection D prime score  CBCL: internalizing and externalizing problems  Wide Range Achievement Test | No significant differences in KABC-II, TOVA, or wide range achievement test scores were identified, though the malaria cohort had worse scores for internalizing behavior problems than community controls (P= 0.007). Borderline differences found in attention. | Fair |

SMA= Severe malarial anemia; BCS= Blantyre Coma Scale; KABC-II= Kaufman Assessment Battery for Children-Second Edition; BRIEF= Behavior Rating Inventory for Executive Function; CBCL= Achenbach Child Behavior Checklist; MDAT= Malawi Developmental Assessment Tool; MSEL= Mullen Scales of Early Learning; TOVA= Test of Variables of Attention; AMD= Adjusted mean difference; RR= Risk ratio; TPT= Tactual Performance Test
